# Underdiagnosis, false diagnosis and treatment of COPD in a selected population in Northern Greece

**DOI:** 10.1080/13814788.2021.1912729

**Published:** 2021-05-24

**Authors:** Dionisios Spyratos, Diamantis Chloros, Dionisia Michalopoulou, Ioanna Tsiouprou, Konstantinos Christoglou, Lazaros Sichletidis

**Affiliations:** a Pulmonary Department, Aristotle University of Thessaloniki, G. Papanikolaou Hospital, Thessaloniki, Greece; b Primary Care Center, Municipality of Thessaloniki, Thessaloniki, Greece; c 3^rd^ Regional Health Care Authority, Thessaloniki, Greece

**Keywords:** Primary care, COPD diagnosis, underdiagnosis, false diagnosis, overtreatment

## Abstract

**Background:**

In the primary care setting, diagnosis and treatment of COPD is not always consistent with GOLD guidelines.

**Objectives:**

To calculate the prevalence of COPD underdiagnosis, false diagnosis and treatment in the general population of northern Greece.

**Methods:**

Observational study in the context of an early COPD detection and smoking cessation project. Inclusion criteria: >40-year-old, current and former smokers (>10 pack-years) in five primary care centres of northern Greece from 2012 to 2019. Participation was achieved *via* a campaign (posters and advertisements in the mass media).

**Results:**

We examined 5,226 subjects (mean age: 58.2 ± 12.7 years, 61.5% males, current smokers: 56.2%) of whom 564 (10.8%) had symptoms and spirometrically confirmed COPD. There were 5 groups of ‘interest:’ **a)** 117/264 (44.3%) with a previous correct diagnosis COPD and correct treatment; **b)** 139/264 (52.7%) previous correct diagnosis COPD but overtreatment; **c)** 8/264 (3%) previous correct diagnosis COPD but undertreatment; **d)** 461 subjects (63.6% of those with previous COPD diagnosis) had previous false diagnosis of COPD (= also overtreatment); **e)** 300/564 (53.2%) previously not diagnosed COPD (=underdiagnosis and also undertreatment). We found that 322/461 (69.8%) of those with a previous false diagnosis have been prescribed long-acting bronchodilators plus ICS.

**Conclusion:**

Among the general population subjects in northern Greece, more than 50% of patients with COPD were underdiagnosed, more than 50% of correctly diagnosed COPD patients were overtreated and most patients taking inhaled drugs were those with a false diagnosis of COPD (possibly GOLD stage 0).

KEY MESSAGES

- In primary care centres in northern Greece,
- >50% of the patients with COPD are underdiagnosed
- >50% of correctly diagnosed COPD patients are overtreated
- Most patients who were taking inhaled drugs in the primary care setting have a false diagnosis of COPD (GOLD stage 0)

## Introduction

Chronic Obstructive Pulmonary Disease (COPD) is a chronic respiratory disease characterised by irreversible airflow limitation. Exacerbations, symptoms and comorbidities are related to its severity [1]. Underdiagnosis of COPD worldwide, especially in the community setting, is a common problem and is estimated between 50 to 80% [2,3]. Parameters related to COPD underdiagnosis are underuse of spirometry, some patients’ characteristics (age, sex, preserved quality of life) and mild obstruction [4]. Overdiagnosis/misdiagnosis (false-positive diagnosis) has been attributed to the spirometric threshold for defining COPD, errors related to spirometry technique, errors made in primary care, differential diagnoses and patient’s demographic characteristics [5]. Additionally, even though pharmacological therapy proved to be an effective intervention management of COPD, it is not always consistent with GOLD guidelines in everyday clinical practice (usually overtreatment) [6–8]. Underdiagnosis deprives patients of effective pharmaceutical and nonpharmaceutical therapies for COPD. On the other hand, a false diagnosis of COPD may lead to unnecessary and even harmful drugs as well as missed detection and treatment of other diseases [9]. In Greece, we have no national guidelines for COPD diagnosis and treatment in primary care setting exclusively and no official training of GPs on respiratory medicine during their residency.

This present observational study aimed to calculate the prevalence of COPD underdiagnosis, false diagnosis and treatment adequacy according to GOLD guidelines 2020 among an unselected sample of general population in northern Greece.

## Methods

### Study design

This was an observational study among unselected subjects of general population conducted in the context of an early COPD detection/smoking cessation project of the 3rd Regional Health Care Authority of Greece and took place from 1/1/2012 to 31/12/2019. General practitioners (GPs) of four counties (rural areas) and doctors from the primary health centre of the metropolitan city of Thessaloniki (urban area, the second biggest city in Greece) collaborated with pulmonologists from Pulmonary Department/Aristotle University of Thessaloniki. Population referred to the above primary health centres is about 500,000 inhabitants. There was no spirometry use in any of these five primary health centres but in the past, many participants had undergone spirometry by pulmonologists or certified GPs. Invitation to the public was done *via* a campaign about respiratory health/early diagnosis of COPD (posters and advertisements in the mass media for the referred geographic region of these five primary care centres) and there was no random representative sampling. Every subject who wanted to follow this COPD detection/smoking cessation programme made an appointment with GPs in the rural areas and with a secretary in the primary health care centre of Thessaloniki. It was a public health prevention programme of the 3rd Regional Health Care Authority of Greece in collaboration with Aristotle University.

### Selection of study subjects – inclusion/exclusion criteria

Participants in the present study were subjects of general population, >40-year-old, current and former smokers (>10 pack-years). All participants were included based only on demographic factors (age, smoking status). Previous medical diagnosis of COPD, treatment with inhaled drugs or the presence of symptoms were inclusion criteria. Study period was 1/1/2012 − 31/12/2019 (when the project was active). Participants with a previous medical diagnosis of respiratory disease other than COPD (e.g. bronchial asthma, bronchiectasis, lung cancer, tuberculosis, interstitial lung disease) were excluded from the statistical analysis.

We should mention that there are no national guidelines in Greece for COPD diagnosis and treatment in primary care setting exclusively and there is no official training of GPs on respiratory medicine during their residency.

### Data collection – measurements

A group of experienced board-certified pulmonologists interviewed all participants, performed and analysed spirometries (MIR SpiroLab II, Roma Italy, CE0476, before and 15–30 min after 400 μg of salboutamol). We collected data about medical history relevant for COPD severity (previous medical diagnosis and treatment for COPD was retrieved from national electronic prescription system, questionnaires about mMRC scale for dyspnoea, CAT score during the last 4 weeks, exacerbations during the last year and comorbidities were reported if the participant was diagnosed with COPD based on spirometry).

### Variables and definitions

Diagnosis of COPD was based on compatible symptoms plus post bronchodilation FEV_1_/FVC < 0.7. COPD patients were categorised based on airflow limitation (GOLD 1: FEV_1_ ≥80%pred., 2: 80 < FEV_1_≥50%pred., 3: 50 < FEV_1_≥30%pred. and 4: <30%pred.) and GOLD guidelines 2020 groups according to exacerbations history during the last year (0-1 and ≥2) and symptoms [we used the worse of score of mMRC (0-1 and ≥2) or CAT (<10 and ≥10) to evaluate GOLD A, B, C and D severity subgroups] [1].

We defined underdiagnosis as the case of a patient with COPD (clinical and spirometric criteria fulfilled) but without a previous official medical diagnosis (we established the diagnosis during this screening program, newly diagnosed patients).

We defined previous false diagnosis as the case of a subject with normal spirometry (pre and post FEV_1_/FVC >0.7, FVC and FEV_1_ >80% of predicted values) during the present study evaluation who wrongly had a previous medical diagnosis of COPD and had been prescribed inhaled drugs for at least 12 consecutive months during the last 5 years (data was retrieved from national electronic prescription system). Participants who were presented with restriction (pre and post FEV_1_/FVC >0.7, FCV and FEV_1_ <80% of predicted values) were referred to pulmonologists for further investigation.

Overtreatment was defined as prescription of an inhaled drug not included in the current GOLD treatment recommendations.

We used descriptive statistics to calculate COPD prevalence (previously correct and newly diagnosed cases) and the prevalence of previous false positive diagnosis.

### Medical research ethics

All participants gave informed consent for retrieving their medical data from the national electronic prescription system. 3rd Regional Health Care Authority of Greece approved study (protocol number approval: 40191, 30/10/2011).

## Results

We examined 5,226 subjects (mean age: 58.2 ± 12.7 years, 61.5% males, median pack-years: 34.1, current smokers: 56.2%) of whom 564 (10.8%) had compatible respiratory symptoms and spirometry that confirmed the diagnosis of COPD according to GOLD guidelines. No participant had a history of hospitalisation for COPD exacerbation during his/her life. There was no missing data as all participants performed acceptable spirometries before and after bronchodilation.

### Diagnosis (underdiagnosis, false diagnosis)

**Figure 1.**
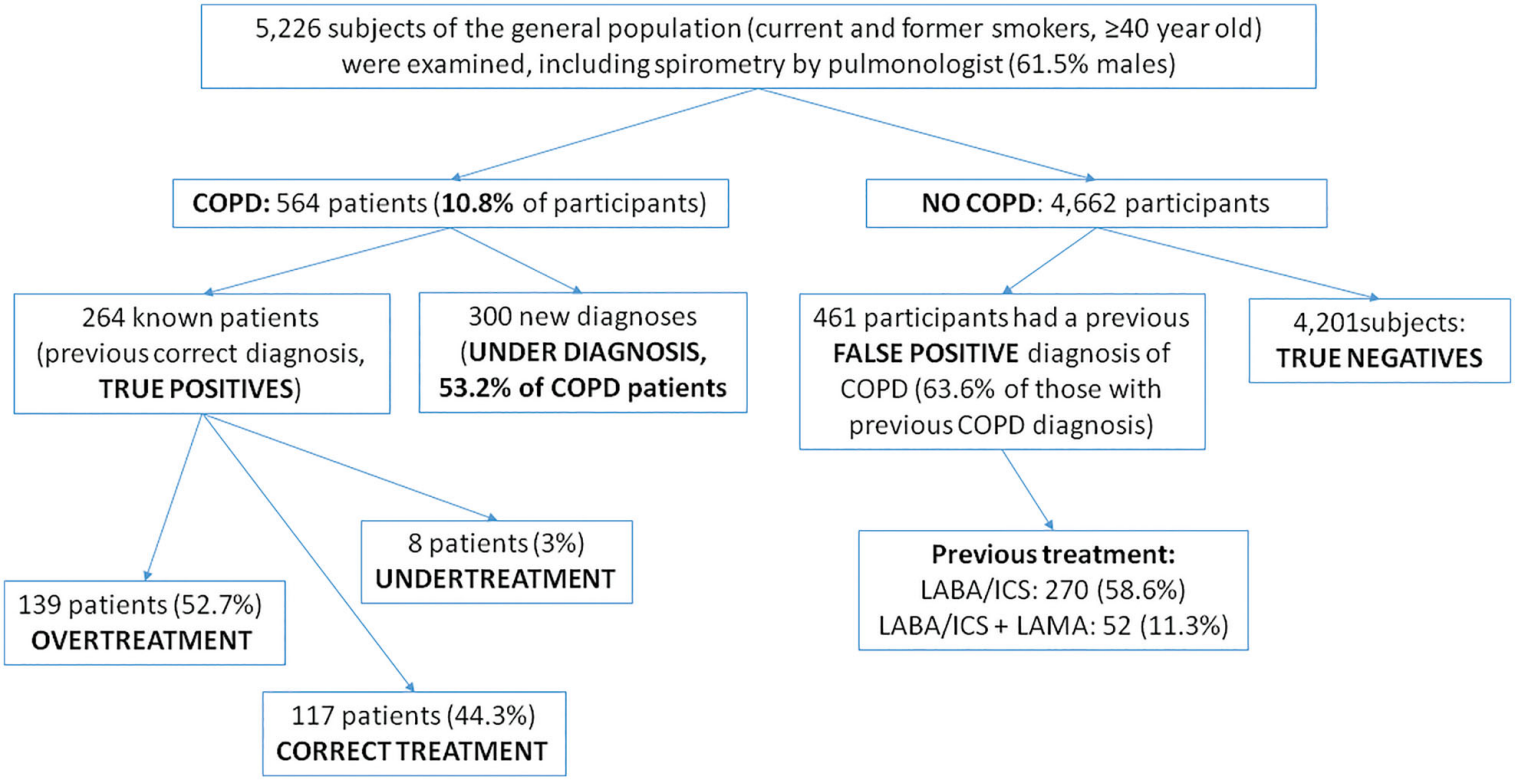
Flow diagram of the study showing the prevalence of under diagnosis, false diagnosis and overtreatment.

**Figure 2.**
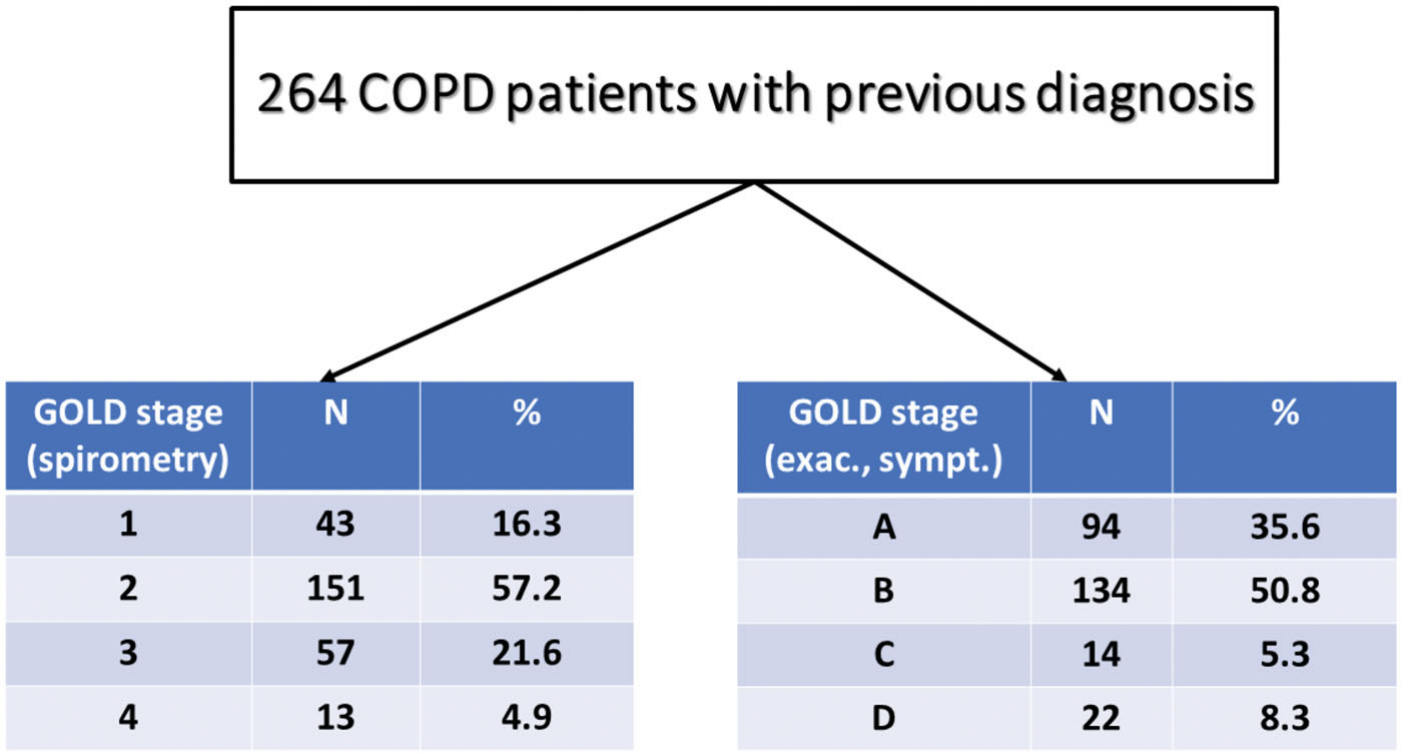
Severity classification of patients with COPD.

Patients with underdiagnosis (newly diagnosed cases) of COPD were 300/564 (53.2%, Figure 1). COPD severity based on spirometry (GOLD 1, 2, 3 and 4) and clinical parameters (groups A, B, C and D) are shown in Figure 2 both for underdiagnosed patients and those with a correct diagnosis in the past. False diagnosis was detected among 461 subjects (8.8%, Figure 1).

### Treatment (overtreatment, undertreatment)

Patients with a previous correct COPD diagnosis who already treated with one or two inhaled long-acting bronchodilators plus inhaled corticosteroids were 164/264 (62%, LABA/ICS: 96, LABA/ICS + LAMA: 68) while taking into account COPD severity, 86.2% of them should have been treated only with long-acting bronchodilators (GOLD A + B, see Figure 2). Based on GOLD 2020 guidelines, we found that 117 patients (44.3%) were treated adequately, 8 (3%) were undertreated and 139 (52.7%) were overtreated.

Considering that 725 participants (264 + 461) had a previous medical diagnosis and treatment of COPD, false diagnosis represents 63.6% of the subjects who were prescribed inhaled drugs for COPD in the primary care setting. For this subgroup of participants, we should mention that COPD diagnosis in the past was based mainly on smoking history and respiratory symptoms and only a minority of them had a previous official spirometry test (stage 0 according to GOLD guidelines 2001). We should also emphasise that 322/461 (69.8%) of those with false diagnosis were treated with LABA/ICS: 270 and LABA/ICS + LAMA: 52. We changed inhaled medications for overtreated patients and stopped treatment for those with false diagnosis.

## Discussion

### Main findings

Out of 5,226 participants, 564 (10.8%) had compatible respiratory symptoms and spirometry that confirmed the diagnosis of COPD according to GOLD guidelines. The present study shows that more than 50% of the patients with COPD were underdiagnosed, overtreatment was observed in more than 50% of patients with a previous correct diagnosis of COPD and most patients on treatment with inhaled drugs in the primary care setting are subjects with respiratory symptoms who are falsely diagnosed and treated as COPD (GOLD stage 0).

### Strengths and limitations

The main limitations of the present study were the absence of random selection sampling of the study population while the primary health care centres that were included are located in five out of thirteen counties of northern Greece. As we analysed data at the end of study period we used current (2020) guidelines as standard for treatment adequacy and not previous versions on the time point of evaluation. No use of spirometry in all five primary care practices of the study may explain the high prevalence of underdiagnosis and misdiagnosis even though all COPD patients with correct diagnosis in the past had undergone official spirometry.

On the other hand, the main advantage of the study is the large number of participants who also represent an everyday clinical practice, primary care population of early COPD (no hospitalisation for COPD exacerbation was reported, 86% of previously and 91% of newly diagnosed patients were categorised as group A + B).

### Interpretation of the study results in relation to existing literature

A recent review on the topic emphasises that only 20-30% of COPD patients have been diagnosed, approximately 70% of them worldwide may be underdiagnosed (53.2% in the present study) and 30-60% of patients with a previous physician diagnosis of COPD do not have the disease (63.6% of those with previous COPD diagnosis in the present study) [3].

Tupper et al., in a recent study among 3,875 smokers (examined by 241 GPs, >5% of Danish GPs) concluded age [10], BMI <25 kg/m^2^ and the presence of symptoms (self-reported dyspnoea, wheeze and sputum) were associated with a significantly higher risk of having underdiagnosed COPD (all underdiagnosed patients in the present study were symptomatic). Canals-Borrajo et al., examined 173 smokers (acceptable spirometry and reversibility testing) in Mallorca/Spain and found that 22.5% had underdiagnosed COPD (89.7% stage I and II) [11]. In our study, the respective percentage was 91.3% stage I and II). Underuse of spirometry in primary care [12], underreporting of relevant symptoms to their physicians and milder disease severity are the main reasons for underdiagnosis [13].

Use of spirometry with correct interpretation of the results can avoid a substantial proportion of cases of false diagnosis of COPD [14]. False positive diagnosis of COPD could be observed in many different ways: normal spirometry excludes COPD (all subjects with misdiagnosis of COPD in the present study had normal spirometry after bronchodilation), postbronchodilation spirometry is necessary, use of specific threshold of FEV_1_/FVC for diagnosis (<0.7 or < LLN, taking into account clinical presentation), some comorbidities (heart failure, severe asthma) may present with fixed airway obstruction and follow-up (with or without treatment) may lead to normal spirometry. In a cross-sectional study from Sweden, only a third of patients with a new diagnosis of COPD had confirmation with spirometry after bronchodilation as proposed by guidelines [15]. In a recent study from Italy among unselected patients in primary care, only 13.3% of doctor-diagnosed COPD patients had concordant spirometric pattern [16].

A recent study from central Greece showed that 61% of non-COPD participants used inhaled medication [8], a conclusion similar to our results. A real-world study in the UK primary care database showed that 53.7% of COPD population was receiving ICS [7]. A similar study about prescribing practices in the UK during the last decade showed that 41% of all COPD patients were maintained on triple therapy while over 1/3 of them suffered from mild disease [17].

GPs usually prescribe drugs based on patients’ symptoms, clinical examination and smoking history while they do not use spirometry in everyday clinical practice for differential diagnosis (obstructive vs restrictive disorders, bronchodilation test). Additionally, even if their diagnosis of COPD is correct, they usually prescribe long-acting bronchodilators plus ICS to cover all the spectrum of the disease severity as well as the case of persistent bronchial asthma. There is an urgent need for improvement on education and training of GPs in Greece on COPD diagnosis (risk factors, symptoms and spirometric criteria) as well as the implementation of national guidelines for primary care that will eventually lead to decrease of overdiagnosis/misdiagnosis, underdiagnosis and inappropriate treatment selections. We should mention that during 2020 a six-month training period on respiratory medicine has been added in the new residence program of GPs in Greece.

## Conclusion

Among current and former smokers (>10 pack-years), >40-year-old of general population in northern Greece, more than 50% of patients with COPD were underdiagnosed, more than 50% of correctly diagnosed COPD patients were overtreated and most patients treated with inhaled drugs in the primary care setting are subjects presented with respiratory symptoms who are incorrectly diagnosed and treated as severe COPD (possibly GOLD stage 0).
